# Knockdown of the long noncoding RNA XIST suppresses glioma progression by upregulating miR-204-5p

**DOI:** 10.7150/jca.45676

**Published:** 2020-05-18

**Authors:** Jun Shen, Jianhua Xiong, Xuefei Shao, Hao Cheng, Xinyun Fang, Yongkang Sun, Guangfu Di, Jie Mao, Xiaochun Jiang

**Affiliations:** 1 Department of Neurosurgery, The First Affiliated Hospital (Yijishan Hospital) of Wannan Medical College, Wuhu, P.R. China; 2 Department of Neurosurgery, Tianjin Medical University General Hospital, Tianjin, 300052, P.R. China; 3 Department of Neurosurgery, Shenzhen Hospital, Southern Medical University, Shenzhen, 518000, P.R. China

**Keywords:** Knockdown, RNA XIST, glioma progression, miR-204-5p

## Abstract

**Background**: Gliomas are the most prevalent primary malignant tumors of the central nervous system. Our previous study showed that miR-204-5p is a tumor suppressor gene in glioma. Bioinformatic analyses suggest that long noncoding RNA (lncRNA) X-inactive specific transcript (XIST) is a potential target gene of miR-204-5p.

**Methods**: We analyzed the expression of XIST and miR-204-5p in glioma tissues and the correlation with glioma grade. A series of in vitro experiments were carried out to elucidate the role of XIST in glioma progression. A mouse xenograft model was established to detect whether knockdown of XIST can inhibit glioma growth. A luciferase assay was performed to determine whether XIST can bind to miR-204-5p and the binding specificity. Cells stably expressing shXIST or shNC were transfected with anti-miR-204-5p or anti-miR-204-5p-NC to evaluate whether XIST mediates the tumor-suppressive effects of miR-204-5p.

**Results**: XIST was upregulated in glioma tissues compared with normal brain tissues (NBTs), while miR-204-5p expression was significantly decreased in glioma tissues compared with NBTs. Both XIST and miR-204-5p expression levels were clearly related to glioma grade, and the expression of XIST was obviously negatively correlated with miR-204-5p expression. Knockdown of XIST inhibited glioma cell proliferation, migration, and invasion, promoted apoptosis of glioma cells, inhibited tumor growth and increased the survival time in nude mice. miR-204-5p could directly bind to XIST and negatively regulate XIST expression. XIST mediated glioma progression by targeting miR-204-5p in glioma cells. XIST crosstalk with miR-204-5p regulated Bcl-2 expression to promote apoptosis.

**Conclusion**: Our results provide evidence that XIST, miR-204-5p and Bcl-2 form a regulatory axis that controls glioma progression and can serve as a potential therapeutic target for glioma.

## Introduction

Glioma is the most prevalent primary malignant tumor of the central nervous system 1. The overall incidence for all gliomas ranges from 4.67 to 5.73 per 100 000 individuals per year 2, 3. The standard treatment includes operation, radiation and temozolomide chemotherapy 4. Despite many research efforts on the pathogenesis and treatment of glioma in the past few decades, the prognosis of glioma patients has not been significantly improved, especially for high-grade gliomas, with a median survival time of only 1-2 years 5. This is mainly because therapeutic resistance and diffuse growth of the glioma tend to recur after surgery 6, 7. Understanding the molecular and cellular alterations involved in glioma progression can help to diagnose, predict prognosis and explore possible treatment strategies 8-10.

MicroRNAs (miRNAs) are small, noncoding RNAs with a length of 22 nucleotides. They regulate the expression of a variety of genes by interacting with the 3'-untranslated regions of their messenger RNA targets. They have been defined as a new type of gene expression regulator that functions by degradation or translational repression 11, 12. Accumulating evidence suggests that miRNAs regulate the malignant biological behavior of multiple cancers, such as non-small cell lung cancer 13, breast cancer 14, pancreatic cancer 15, hepatocellular carcinoma 16, colon cancer 17, and glioma 18, 19.

Human miR-204-5p has been demonstrated to be a tumor suppressor gene that is involved in the molecular and biological processes of various types of cancers 20-26. Our previous research showed that miR-204-5p was downregulated in both glioma tissues and glioma cell lines, and miR-204 mimics significantly decreased glioma cell migration and invasion 27. Further bioinformatic analyses (miRcode (website: www.mircode.org), starBase 2.0 (website: http://starbase.sysu.edu.cn/)) suggest that long noncoding RNA (lncRNA) X-inactive specific transcript (XIST) is a potential target gene of miR-204-5p. XIST is derived from the XIST gene and initially identified by Brockdorff in 1991 28. A previous study demonstrated that XIST was upregulated in glioma tissues, and knockdown of XIST inhibited glioma cell migration, invasion and proliferation and promoted apoptosis of glioma cells 29.

Therefore, in this study, we first detected the expression of XIST and miR-204-5p and their correlation in glioma tissues and normal brain tissues (NBTs). Then, we determined whether XIST regulated the malignant biological behavior of glioma cells by targeting miR-204-5p.

## Materials and Methods

### Human tissue samples

Ten normal brain tissues (NBTs) and fifty-five primary glioma tissues were collected from patients undergoing surgery at the Department of Neurosurgery, The First Affiliated Hospital (Yijishan Hospital) of Wannan Medical College from 2015 to 2017. All tissues were immediately frozen in liquid nitrogen after resection for subsequent analysis. Each patient or their family members signed an informed consent form. This study was carried out in compliance with the Helsinki Declaration and was approved by the Institutional Ethical Review Committee of The First Affiliated Hospital (Yijishan Hospital) of Wannan Medical College. The grading of tumors was confirmed by pathologists according to the WHO classification criteria. None of the patients were concomitant with cancer of other systems and had not previously received radiotherapy and/or chemotherapy.

### Cell lines and cell culture

The human U87 and U251 cell lines were obtained from RuanTuo Biotechnology Co. Ltd. (Shanghai, China). Cells were cultured in Dulbecco's modified Eagle medium (DMEM, Gibco, USA) containing 10% fetal bovine serum (FBS, Gibco, USA) and penicillin-streptomycin antibiotics and placed in an incubation chamber with 5% CO2 at 37°C.

### Lentivirus construction and infection

Lentiviral short hairpin RNA targeting XIST and a negative control were designed and ligated with the LV10-CMV-RFP-Puro vector (GeneChem, Shanghai, China). The lentiviral particles were harvested 72 hours after packaging in HEK293T cells. The lentivirus and control virus were fluorescently labeled and then infected with glioma cells. Cells stably expressing lentivirus were identified as shXIST or shNC for subsequent experiments.

### Cell transfection

Transfection was carried out by using Lipofectamine 2000 (Invitrogen, USA) according to the manufacturer's instructions. MiR-204-5p agomir, miR-204-5p antagomir and their negative control (NC) were purchased from GeneChem, Shanghai, China. Transfected cells were divided into 5 groups: control group, pre-miR-204-5p-NC group, pre-miR-204-5p group, anti-miR-204-5p-NC group and anti-miR-204-5p group. The shXIST and shNC stably expressing cells cotransfected with miR-204-5P antagomir or their negative control were divided into five groups: control group, shNC + anti-miR-204-5p-NC group (shNC stably expressing cells cotransfected with anti-miR-204-5p-NC), shNC + anti-miR-204-5p group (shNC stably expressing cells cotransfected with anti-miR-204-5p), shXIST + anti-miR-204-5p-NC group (shXIST stably expressing cells cotransfected with anti-miR-204-5p-NC), and shXIST + anti-miR-204-5p group (shXIST stably expressing cells cotransfected with anti-miR-204-5p).

### RNA extraction and quantitative real-time RT-PCR

Total RNA of cell lines or tissue samples was extracted by using TRIzol reagent (Life Technologies, USA) according to the manufacturer's instructions. Total RNA was reverse transcribed to cDNA by Bulge-Loop miRNA (RiboBio, Guangzhou, China) following the provided protocol. Then, qPCR was carried out using SYBR Master Mix (TAKARA, Dalian, China). GAPDH was used as an endogenous control. The relative expression levels of the target genes were normalized to GAPDH levels and calculated as the fold change (2^-ΔΔCt^ method). The primers used were as follows: XIST forward, 5'-GTGGGATGTTGCTAACGAGTGC-3' and reverse, 5'-TGCTGGAATACAAAGGGAGTGG-3'; miR-204 forward, 5'-CTGTCACTCGAGCTGCTGGAATG-3' and reverse, 5'-ACCGTGTCGTGGAGTCGGCAATT-3'; GAPDH forward, 5'-TGACTTCAACAGCGACACCCA-3' and reverse, 5'-CACCCTGTTGCTGTAGCCAAA-3'.

### Cell proliferation assay

A CCK-8 assay (Biyuntian, Shanghai, China) was used to detect the cell proliferation ability. Briefly, cells (2000 cells/well) were seeded into 96-well plates and cultured for 24 hours prior to experiments at 37°C. CCK-8 solution (10 *μ*l/well) was added to the plates. After 2 hours of incubation, the spectrophotometric absorbance at 450 nm was measured as the initial proliferation at day 1, and then the absorbance at 450 nm was measured every 24 hours for 4 consecutive days.

### Cell apoptosis assay

After 3 days of infection with lentivirus, cells were seeded in 6-well plates (5×10^5^ cells/well) and incubated for 24 hours until the density of cells exceeded 85%. Cells were washed, resuspended, and then stained with 10 μL Annexin V-APC (22838, ATTBioquest, USA) per well. Cells were analyzed by flow cytometry (Millipore, Guava, France).

### Western blot analysis

Cells were harvested and lysed by RIPA lysis buffer (1% Triton X-100, 0.1% SDS, 10 mM Tris-HCl, 1 mM MgCl2, pH 7.4) for 30 minutes at 4°C. The extracted protein was separated by SDS-PAGE and transferred onto PVDF membranes. The membranes were blocked with 5% BSA in TBS-T for 1 hour at room temperature and then incubated with anti-Bcl-2 (1:1000) and anti-β-actin (1:1000) antibodies in 3% BSA overnight at 4°C. After one hour of incubation with secondary antibodies, the membranes were submerged with developer for exposure. The following reagents and antibodies were used: anti-Bcl-2 antibody (Proteintech, USA); anti-β-actin antibody (CST, USA); PVDF membranes (Millipore, Bedford, MA); RIPA buffer (Biyuntian, Shanghai, China); and BCA Protein Assay Kit (Biyuntian, Shanghai, China). The integrated density values were measured using Image J software (NIH, Bethesda, MD, USA).

### Cell migration and invasion assays

Transwell and wound healing assays were used to evaluate cell migration and invasion. For the transwell assay, 24-well transwell chambers with a pore size of 8 mm polycarbonic membrane (Corning, NY, USA) were used. Cells (1×10^5^/well) were seeded into the upper chambers with serum-free DMEM, while 600 µL DMEM containing 30% FBS was added into the lower chambers. After incubation at 37°C for 24 hours, cells in the upper chambers were removed, and cells in the lower chambers were fixed with 4% paraformaldehyde and stained with crystal violet. The number of cells in the lower chambers was counted under a microscope (Caikang, Shanghai, China). Cells (1×10^5^/well) were transferred to 12-well plates to perform the wound healing assay. After wounds were created by a 10 *μ*l plastic pipette tip, the cells were washed and cellular debris was removed, and the cells were incubated at 37°C for 24 hours. The wound area at 0 hours and wound healing area at 24 hours were measured by Image J software (NIH, Bethesda, MD, USA).

### In vivo xenograft experiments

Animal experiments were performed in accordance with the protocols approved by the Animal Care Committee of The First Affiliated Hospital (Yijishan Hospital) of Wannan Medical College. Four-week-old female athymic BALB/c nude mice were purchased from Shanghai Lingchang Biological Technology Co., Ltd. (Shanghai, China) and were maintained under pathogen-free conditions. To analyze tumor propagation, 5 × 10^6^ U87 cells stably expressing shXIST or shNC were subcutaneously injected into animals. Tumor volume was measured every three days and calculated using the following formula: volume (mm^3^) = length × width× width/2. Cell inoculation and tumor growth were detected using an in vivo imaging system (Perkin Elmer, USA). The experiment was terminated at 24 days, mice were harvested, and tumor weights were measured. For animal survival analysis, 5 × 10^6^ U87 cells stably expressing shXIST or shNC were stereotactically implanted into the right striatum of each mouse The numbers of dead and surviving nude mice were recorded.

### Luciferase reporter assay

For the luciferase reporter assay, the wild-type or mutant putative sequences of the binding site of XIST were cloned into the pmirGLO Dual-luciferase Vector (Promega, USA) to form the reporter vector pmirGLO-XIST-wild-type (XIST-Wt) or pmirGLO-XIST-mutant (XIST-Mut). XIST-Wt or XIST-Mut was cotransfected with miR-204-5p mimics or negative control into glioma cells using Lipofectamine 2000. The firefly luciferase activity of each group was assayed 48 hours after transfection using a Dual Luciferase Reporter Assay System (Promega, USA) and normalized to Renilla luciferase activity following the manufacturer's protocol.

### Statistical analysis

All data are presented as the mean ± standard deviation (SD) from three independent experiments. Differences between two groups were analyzed by using Student's t-test, and the differences between three or more groups were analyzed by using one-way ANOVA (LSD and Bonferroni correction). Linear regression was used to analyze the correlation between miR-204-5p and XIST expression in tissues. Animal survival analysis was performed by using the Kaplan-Meier survival curve. All statistical analyses were carried out by using SPSS 22.0 software (IBM, New York, USA), and a *p*-value less than 0.05 was considered statistically significant.

## Results

### XIST expression was upregulated in glioma tissues, while miR-204-5p was downregulated in glioma tissues

**Fig 1:**
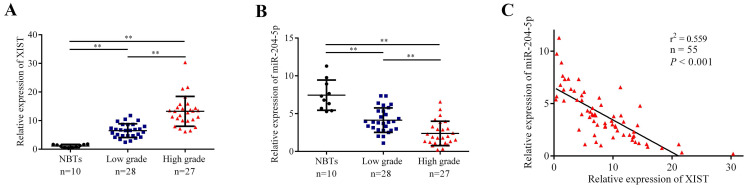
The expression of XIST and miR-204-5p in human normal brain tissue (NBTs) and glioma tissues. A and B: The expression of XIST and miR-204-5p in NBTs and glioma tissues of different grades were evaluated by qRT-PCR. The relative expression levels were normalized to GAPDH as fold change. Data are presented as the mean ± SD (NBTs (n = 10), Low grade (n = 28), High grade (n = 27)). **P < 0.01. C: The correlation between miR-204-5p and XIST expression in glioma tissues was assessed by using Linear regression. The expression of XIST was obviously negative correlated with the miR-204-5p expression. r^2^ = 0.559, *P* < 0.001.

The expression levels of XIST and miR-204-5p in human NBTs and glioma tissues were evaluated by qRT-PCR. As shown in Fig. 1A and 1B, XIST expression was significantly increased in glioma tissues of different pathological grades compared with NBTs, and the expression was elevated along with the pathological grades, while miR-204-5p expression was significantly decreased in glioma tissues compared with NBTs, and the expression level was strongly correlated with the tumor grade. Moreover, the expression of XIST was obviously negatively correlated with miR-204-5p expression (Fig. 1C).

### Knockdown of XIST inhibited proliferation and promoted apoptosis of glioma cells

**Fig 2:**
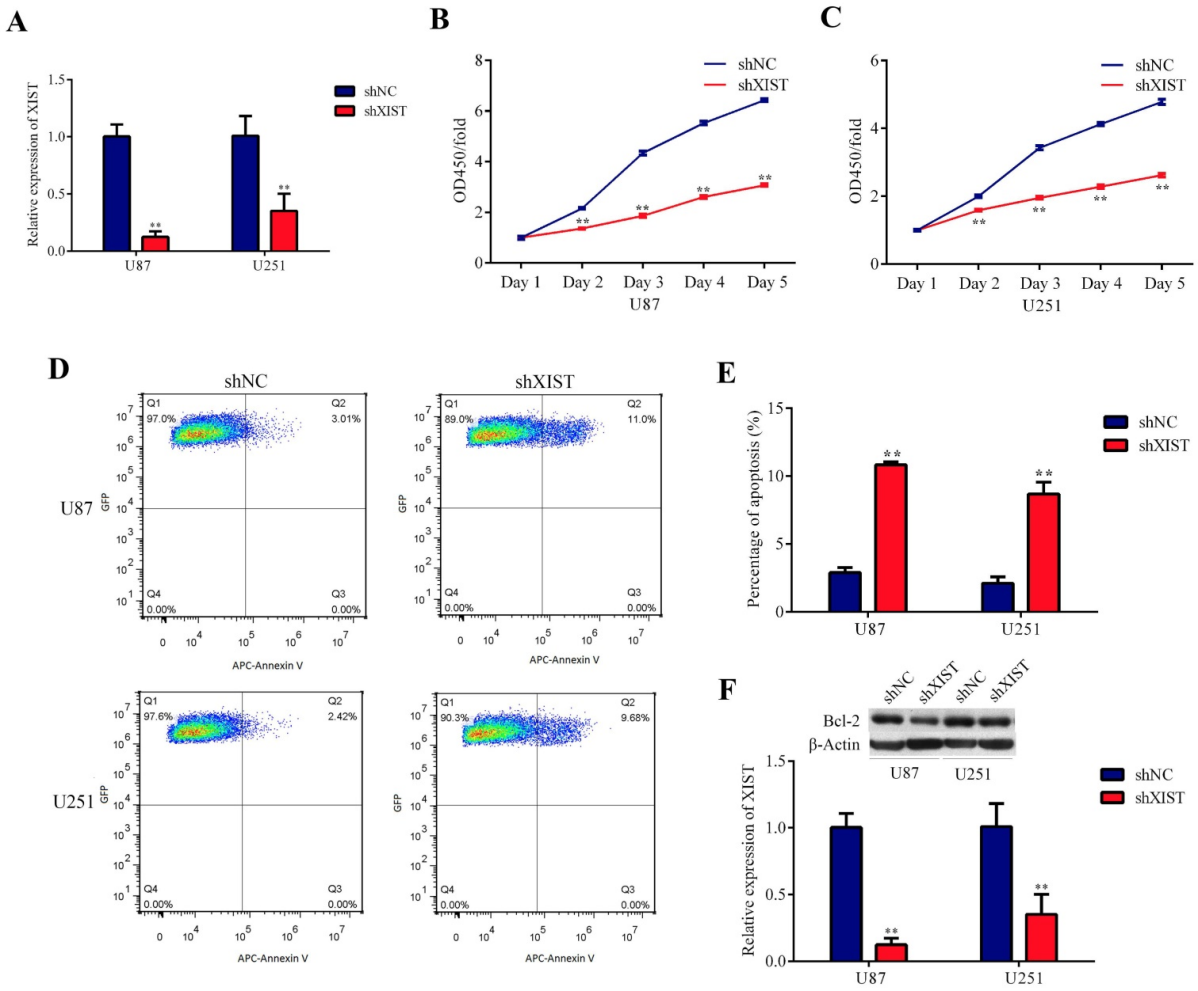
Effect of XIST knockdown on glioma cell proliferation and apoptosis. A: Relative expression of XIST was evaluated by qRT-PCR at 72 hours after the plasmids of shXIST or shNC transfected into U87 and U251 cell lines, the relative expression levels were normalized to GAPDH as fold change. Data are presented as the mean ± SD in three independent experiments. **P < 0.01. B and C: Knockdown of XIST inhibited cell proliferation as indicated by CCK-8 assays in U87 and U251 cells. Proliferation rate are calculate as OD450 fold change to day 1 and presented as the mean ± SD in three independent experiments. **P < 0.01. D: Flow cytometry was employed to evaluate the glioma cell apoptosis, representative images of apoptosis in U87 and U251 cells. E: Knockdown of XIST promoted apoptosis of glioma cells. Data are presented as the mean ± SD in three independent experiments. **P < 0.01. F: Western blot assays revealed that knockdown of XIST decreased the expression of Bcl-2. Data are presented as the mean ± SD in three independent experiments. **P < 0.01.

Because the expression level of XIST was significantly upregulated in glioma tissues, we explored the possible biological role of XIST in glioma progression. Plasmids containing shXIST or shNC were constructed and transfected into U87 and U251 cell lines. The knockdown efficiency is shown in Fig. 2A. The CCK-8 assay revealed that the cell proliferation rate of the shXIST group was significantly reduced compared with that of the shNC group at different time points in both U87 and U251 cells (Fig. 2B and Fig. 2C). Flow cytometric analysis was performed to detect the apoptosis of glioma cells. After shXIST transfection, the apoptosis rate of both U87 and U251 cells was obviously increased compared with that of the shNC transfection group (Fig. 2D and Fig. 2E). XIST has been demonstrated to mediate apoptosis by regulating Bcl-2 expression in both non-small cell lung cancer and cervical cancer 30, 31, and Bcl-2 has also been confirmed to be a direct target of miR-204-5p 32. Therefore, the expression of Bcl-2 was evaluated. Bcl-2 expression was significantly lower in the shXIST group than in the shNC group in both U87 and U251 cells (Fig. 2F), suggesting that knockdown of XIST mediated glioma cell apoptosis via the Bcl-2 pathway.

### Knockdown of XIST inhibited cell migration and invasion

**Fig 3:**
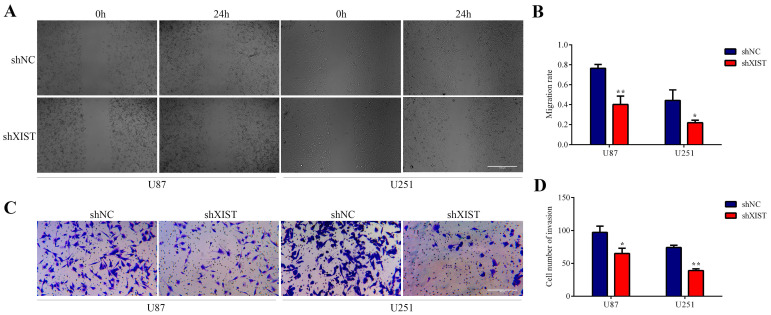
Effect of XIST knockdown on glioma cell migration and invasion. A: Representative images of Wound healing tests in U87 and U251 cells at 0 hour and 24 hours. Scale bars represent 100 μm. B: The migration rate was calculated as wound healing area at 24 hours/ wound healing area at 0 hour. Data are presented as the mean ± SD in three independent experiments. *P < 0.05, **P < 0.01. C: Representative images of Transwell assays in U87 and U251 cells. Scale bars represent 40 μm. D: The number of cells in the lower chambers was counted and represent the migration and invasion abilities. Data are presented as the mean ± SD in three independent experiments. *P < 0.05, **P < 0.01.

In the wound healing tests, the migration and invasion abilities of the shXIST group were significantly inhibited compared with those of the shNC group in both U87 and U251 cells (Fig. 3A and Fig. 3B). The results of the transwell tests were similar to those of the wound healing tests, and the migration and invasion abilities of the shXIST group were significantly decreased compared with those of the shNC group in both U87 and U251 cells (Fig. 3C and Fig. 3D). Knockdown of XIST promoted apoptosis of glioma cells and inhibited cell proliferation, migration and invasion. These results indicated that knockdown of XIST exerted tumor-suppressive functions in glioma cells.

### Knockdown of XIST inhibited tumor growth and prolonged the survival time in nude mice

**Fig 4:**
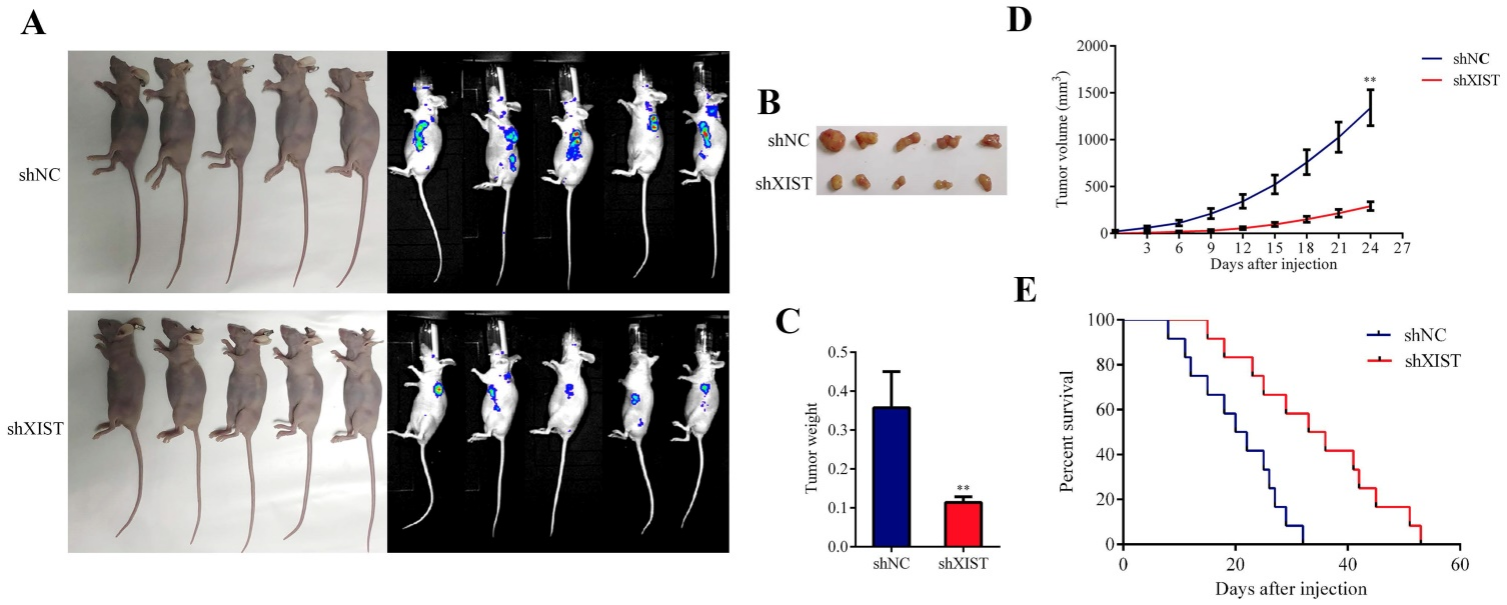
Effect of XIST knockdown on tumor growth survival time in nude mice. A and B: Representative images of the nude mice carrying tumors and vivo imagings of each group (n=9 each group). B: Representative images of the tumors of each group (n=9 each group). C: Tumor volume was measured every three days and calculated using the following formula: volume (mm3) = length × width× width/2 (Data are presented as the mean ± SD, n=9 each group). **P < 0.01. D: Mice were harvested at 24 days and tumor weights were measured (Data are presented as the mean ± SD, n=9 each group). **P < 0.01. E: Animal survival analysis was performed by using the Kaplan-Meier survival curve. Knockdown of XIST exhibited longer survival time (n=12 each group). (*P* < 0.01).

To further confirm the tumorigenic effects of XIST in vivo, we established a mouse xenograft model by subcutaneous injection of U87 cells stably expressing shXIST into the left axilla, while U87 cells stably expressing shNC were injected into the left axilla as a control (Fig. 4A and Fig. 4B). As shown in Fig. 4C and 4D, both the tumor weight and tumor volume in the shXIST group were significantly lower than those in the shNC group. The survival analysis revealed that mice in the shXIST group exhibited longer survival times than those in the shNC group (Fig. 4E).

### Regulatory relationship between XIST and miR-204-5p

**Fig 5:**
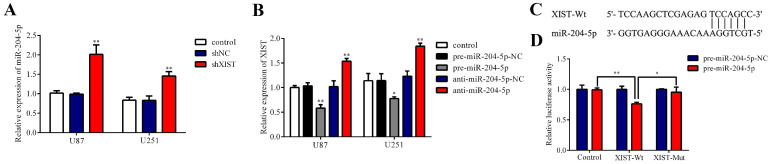
Regulatory relationship between XIST and miR-204-5p. A: Relative expression of miR-204-5p was evaluated by qRT-PCR. Data are presented as the mean ± SD in three independent experiments. vs. control group and shNC group, **P < 0.01. B: Relative expression of XIST was evaluated by qRT-PCR. Data are presented as the mean ± SD in three independent experiments. vs. control group, *P < 0.05, **P < 0.01. C: miR-204-5p binding sequence with XIST-Wt and the sequence of XIST-Mut. D: The firefly luciferase activity of each group was assayed 48 hours after transfection and normalized to Renilla luciferase activity. *P < 0.05, **P < 0.01.

Because bioinformatics analysis suggested that XIST may be a target gene regulating miR-204-5p, qRT-PCR verified that the expression of XIST was negatively correlated with miR-204-5p expression in glioma tissues, and we further investigated whether miR-204-5p is negatively regulated by XIST in glioma cells. We first detected the expression of miR-204-5p in the control group, shNC group and shXIST group. As shown in Fig. 5A, the expression of miR-204-5p was significantly increased in the shXIST group compared with the control group and shNC group in both U87 and U251 cells. Next, we analyzed whether miR-204-5p regulates XIST by determining the effects of miR-204-5p inhibition and overexpression on the expression of XIST. In both U87 and U251 cells, XIST expression was significantly decreased in the pre-miR-204-5p group compared with the control group and pre-miR-204-5p-NC group, while XIST expression was upregulated in the anti-miR-204-5p group compared with the control group and anti-miR-204-5p-NC group (Fig. 5B). To further verify whether XIST is a functional target of miR-204-5p, we cloned the predicted miR-204-5p binding sequences of XIST (XIST-Wt) into the pmirGLO luciferase vector. Cotransfection of pre-miR-204-5p and XIST-Wt significantly decreased luciferase activity, while cotransfection of pre-miR-204-5p-NC and XIST-Wt did not alter luciferase activity, suggesting that XIST was a target of miR-204-5p. To verify the binding site specificity, a mutant miR-204-5p target binding sequence of XIST (XIST-Mut) was cloned into the pmirGLO luciferase vector as a control. Cotransfection of pre-miR-204-5p and XIST-Mut did not alter the luciferase activity (Fig. 5C). Together, these results demonstrate that miR-204-5p can directly bind to XIST and negatively regulate XIST expression.

### XIST mediated the tumor-suppressive effects of miR-204-5p

**Fig 6:**
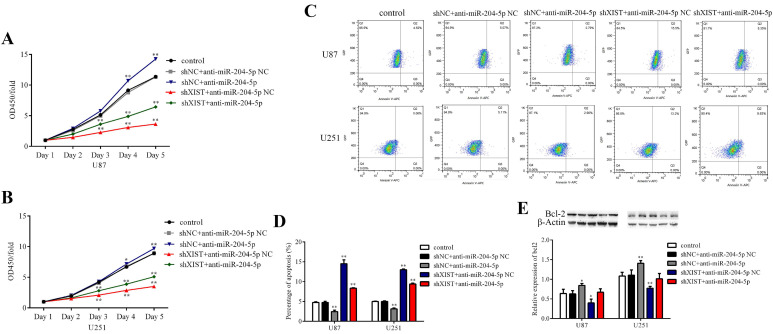
XIST mediated the effect of miR-204-5p on cell proliferation and apoptosis. A and B: Cell proliferation rate of each group were detected by CCK-8 assays and calculate as OD450 fold change to day 1. Date are presented as the mean ± SD in three independent experiments. *P < 0.05, **P < 0.01. C: Representative images of glioma cell apoptosis in each group. D: Effect of XIST knockdown and miR-152 inhibition on glioma cell apoptosis. Date are presented as the mean ± SD in three independent experiments. vs. control group, **P < 0.01. E: Effect of XIST knockdown and miR-152 inhibition on expression of Bcl-2. Date are presented as the mean ± SD in three independent experiments. vs. control group, *P < 0.05, **P < 0.01.

**Fig 7:**
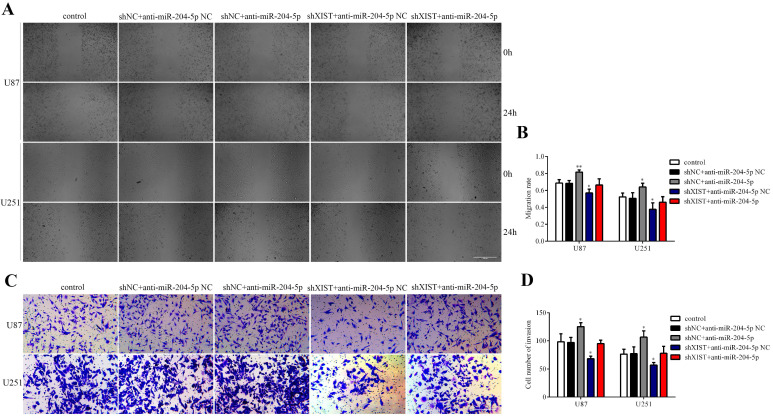
XIST mediated the effect of miR-204-5p on glioma cell migration and invasion. A: Representative images of Wound healing tests in each group. Scale bars represent 100 μm. B: The migration rate was calculated as wound healing area at 24 hours/ wound healing area at 0 hour. Data are presented as the mean ± SD in three independent experiments. Wound healing tests indicated that migration and invasion abilities of glioma cells were increased in shNC+anti-miR-204-5p group but inhibited in shXIST group anti-miR-204-5p NC group. No significant differences was observed in shXIST group anti-miR-204-5p and control group. vs. control group, *P < 0.05, **P < 0.01. C: Representative images of Transwell assays in U87 and U251 cells. Scale bars represent 40 μm. D: The number of cells in the lower chambers was counted and represent the migration and invasion abilities. Data are presented as the mean ± SD in three independent experiments. Transwell assays revealed that knockdown of XIST inhibited migration and invasion of glioma cells, while inhibition of miR-204-5p increased the migration and invasion of glioma cells. vs. control group, *P < 0.05, **P < 0.01.

To determine whether XIST could reverse the tumor suppressive effects of miR-204-5p, cells stably expressing shXIST or shNC were transfected with anti-miR-204-5p or anti-miR-204-5p-NC, and cell proliferation, cell apoptosis, migration and invasion were assessed. As shown in Fig. 6A and 6B, compared with that in the control group and shNC+anti-miR-204-5p NC group, the proliferation rate was obviously reduced in the shXIST+anti-miR-204-5p NC group and shXIST+anti-miR-204-5p group but was increased in the shNC+anti-miR-204-5p group. Flow cytometric analysis revealed that the apoptosis rate in the shXIST+anti-miR-204-5p NC group and shXIST+anti-miR-204-5p group was increased compared with that in the control group and shNC+anti-miR-204-5p NC group but was decreased in the shNC+anti-miR-204-5p group compared with the control group and shNC+anti-miR-204-5p NC group (Fig. 6C and Fig. 6D). We also evaluated the XIST-mediated effect of miR-204-5p on Bcl-2 expression. Compared with that in the control group and shNC+anti-miR-204-5p NC group, Bcl-2 was upregulated in the shNC+anti-miR-204-5p group but downregulated in the shXIST+anti-miR-204-5p NC group (Fig. 6E). Wound healing and transwell assays showed that the migration and invasion abilities in the shNC+anti-miR-204-5p group were significantly increased compared with those in the control group and shNC+anti-miR-204-5p NC group but were significantly decreased in the shXIST+anti-miR-204-5p NC group (Fig. 7A-D). These data suggested that knockdown of XIST suppressed the malignant behaviors of glioma cells by downregulating miR-204-5p. XIST crosstalk with miR-204-5p mediated glioma cell apoptosis via the Bcl-2 pathway.

## Discussion

In this study, we demonstrated that XIST was upregulated in glioma tissues compared with NBTs, and XIST expression was elevated along with the pathological grade, while miR-204-5p expression was significantly decreased in glioma tissues compared with NBTs, and the expression level was negatively correlated with XIST expression. Knockdown of XIST inhibited glioma cell proliferation, migration, and invasion and promoted apoptosis of glioma cells. Knockdown of XIST inhibited tumor growth and prolonged the survival time in nude mice. miR-204-5p was found to directly bind to XIST and negatively regulate XIST expression. Knockdown of XIST suppressed the malignant behaviors of glioma cells by downregulating miR-204-5p. XIST crosstalk with miR-204-5p mediated glioma cell apoptosis via the Bcl-2 pathway.

Previous studies have characterized miR-204-5p as a tumor suppressor gene involved in the molecular and biological processes of various types of cancers. For example, in melanoma, lung cancer and gastric cancer, the expression of miR-204-5p was significantly decreased in tumor tissue and tumor cells, and overexpression of miR-204-5p inhibited the migratory, proliferative and invasive capabilities of tumor cells 20-22, 25. This study combined with our previous results demonstrated that miR-204-5p has a similar effect in gliomas as other tumors 27.

LncRNAs are a class of noncoding RNAs with a length of over 200 nucleotides that lack protein-coding ability 33. Mounting evidence suggests that lncRNAs play an important role in the occurrence and invasion of gliomas, and various lncRNAs and their downstream signaling pathways have been identified to be involved in the proliferation, apoptosis, invasion and migration of glioma cells 34-37. The functions of lncRNAs in tumors are mainly achieved by regulating miRNAs, and miR-204-5p is downregulated in glioma tissues and can inhibit glioma cell migration and invasion. Therefore, we used bioinformatics analysis to identify possible lncRNAs that may regulate miR-204-5p. In this study, XIST was identified as a possible target gene to modulate miR-204-5p in glioma. Our results confirmed that XIST bound to miR-204-5p in glioma cells in a site-specific manner.

Accumulating evidence shows that XIST functions as an oncogene accelerating tumor progression and metastasis in various cancers. Zhang et al demonstrated that XIST was significantly overexpressed in neuroblastoma tissues and cell lines, and knockdown of XIST suppressed the migration and invasion of neuroblastoma cells and inhibited tumor growth 38. Chen et al showed that XIST was upregulated in esophageal cancer tissues and cells. Knocking out XIST significantly promoted apoptosis and inhibited the proliferation, migration and invasion of tumor cells 39. In gastric cancer, overexpression of XIST was significantly associated with larger tumor size, distant metastasis, lymph node invasion, and TNM stage of patients 40. In addition, the expression level of XIST could also be used as a prognostic biomarker for cancer patients 41, 42. However, a recent study may overturn our understanding of the role of XIST in tumors. Xing provided evidence that silencing of XIST preferentially promoted brain metastatic growth of XIST^high^ cells in a xenograft model, and knockdown of XIST in the mammary glands of mice accelerated primary tumor progression and brain metastases 41. This suggests that XIST may play different roles as a tumor suppressor gene or oncogene in different tumor tissues.

In the present study, we demonstrated that knockdown of XIST inhibited glioma cell proliferation, migration, and invasion, promoted apoptosis of glioma cells, inhibited tumor growth and extended survival time in nude mice, indicating that XIST is an oncogene in glioma. These results were consistent with previous research. Yao elucidated that knockout of XIST exerted tumor suppressive functions by inhibiting glioblastoma stem cell proliferation, migration and invasion as well as promoting apoptosis. In a nude mouse model, knockdown of XIST suppressed tumor growth and prolonged survival time 29. In addition, other roles of XIST in glioma have also been discovered, such as increasing blood-tumor barrier permeability, promoting glioma angiogenesis and regulating glucose metabolism in glioma cells 42, 43.

Moreover, we discovered that XIST crosstalk with miR-204-5p mediates glioma cell apoptosis via the Bcl-2 pathway. XIST has been reported to promote tumor cell apoptosis through a variety of apoptosis-related genes, such as Bcl-2, Bax, caspase 3 and caspase 9 30, 31, 44, 45. Among these genes, only Bcl-2 has been reported to be a direct target of miR-204-5p 32; therefore, we evaluated Bcl-2 expression. However, we have not yet determined whether other apoptosis-related genes are involved in this process.

The interactions between lncRNAs and miRNAs are not one-to-one. One lncRNA can regulate multiple miRNAs, and one miRNA can also be regulated by multiple lncRNAs. For example, in addition to miR-204-5p, XIST can also control miR-494, miR-137 and miR-126 to promote glioma progression 29, 42, 43. miR-204-5p has also been reported to be regulated by multiple lncRNAs in human tumors, such as BRAF-activated noncoding RNA and XIST 20. Furthermore, multiple cellular signaling pathways are also involved in the network of lncRNA and miRNA interactions. Therefore, the complex interaction network between lncRNAs and miRNAs and their downstream cellular signaling pathways in the occurrence and progression of glioma deserves in-depth investigation.

## Conclusion

In summary, our results demonstrated that XIST acts as an oncogene that is upregulated in glioma, whereas miR-204-5p was downregulated in glioma tissues and acts as a tumor suppressor gene. XIST mediated glioma progression by targeting miR-204-5p in glioma cells. XIST crosstalk with miR-204-5p mediates glioma cell apoptosis via the Bcl-2 pathway. These results indicate that XIST, miR-204-5p and Bcl-2 form a regulatory axis that controls glioma progression and can serve as a potential therapeutic target for glioma.
